# Supplementary material for: Three Logistic Predictive Models for the Prediction of Mortality and Major Pulmonary Complications after Cardiac Surgery
Source: Medicina (Kaunas). 2023 Jul 26;59(8):1368. doi: 10.3390/medicina59081368 (PMC10456464; doi:10.3390/medicina59081368)
Supplement: Supplementary file 1 [file medicina-59-01368-s001.zip › medicina-2411271-supplementary.pdf]

Supplementary material 1: Definition of postoperative pulmonary complications (PPCs)

Complication	Definition
Respiratory failure	At least one of the following criteria:<br>● SpO_2 < 90% with need for oxygen therapy<br>● PaO_2/FiO_2 < 300 or PaO2 < 60 at room air<br>● $PaCO_2$ > 45 mmHg<br>● Dyspnea with respiratory distress or use of accessory muscles
Respiratory infection	Antibiotic therapy given for a suspected respiratory infection which matches one or more of the following criteria: new onset or modified sputum, new-onset or modified lung opacities, fever, white blood cell count > $12*10^{-9}$/l
Pneumonia	Two or more consecutive chest radiographs with at least one of the following:<br>• new or worsening and persistent infiltrates<br>• consolidation<br>• cavitation;<br>*AND at least one of the following*<br>• fever (>38 °C) with no other acknowledged the cause<br>• leukopenia (white cell count < $4*10^{-9}$/1) or leucocytosis (white cell count >$12*10^{-9}$/1)<br>• altered mental status without other possible cause in adults > 70 years old;<br>*and at least two of the following*<br>• new onset of purulent sputum or modification in the character of sputum, or increased respiratory secretions, or increased suctioning requirements<br>• new onset or worsening cough, or dyspnoea, or tachypnoea<br>• rales or bronchial breath sounds

	• worsening gas exchange (hypoxemia, increased oxygen requirement, increased ventilator demand).
Cardiogenic pulmonary edema	Fluid accumulation in the alveoli due to poor cardiac function.
Pleural effusion	Chest radiograph demonstrating blunting of the costophrenic angle, loss of sharp silhouette of the ipsilateral hemidiaphragm in the upright position, evidence of displacement of adjacent anatomical structures or (in supine position) a hazy opacity in one hemithorax with preserved vascular shadows
Atelectasis	Lung opacification with a shift of the mediastinum, hilum, or hemidiaphragm toward the affected area, and compensatory over-inflation in the adjacent non-atelectatic lung
Aspiration pneumonitis	Inhalation of gastric content in the perioperative period with subsequent acute lung injury
Bronchospasm	Newly detected expiratory wheezing requiring treatment
Pneumothorax	Air in the pleural space

List of abbreviations used

SpO_2: Peripheral Oxygen Saturation (%) – PaO_2: arterial oxygen partial pressure – FiO_2: inspired oxygen fraction – $PaCO_2$: arterial carbon dioxide partial pressure.

Supplementary material 2: Definition of standard intraoperative and postoperative management

A routine pre-defined protocol was applied for perioperative management. Preoperative medications were not administered on the day of surgery. All patients received premedication with morphine 0.1 mg/kg subcutaneously, and intramuscular atropine 0.5 mg one hour before surgery. Anesthesia was induced with fentanyl (5-10 µg/kg), rocuronium (0.6-1.2 mg/kg) and propofol (1-2 mg/kg), or both midazolam and propofol. Anesthesia maintenance was based on fentanyl (3-5 µg/kg/h in repeated boluses before the most algogenic surgical times, such as a sternotomy or pericardiotomy), rocuronium (10 µg/kg/min in continuous infusion), and a combination of sevoflurane, or desflurane and propofol. Tranexamic acid was administered as a continuous infusion to all patients during surgery: a bolus of 1g in 20 minutes followed by a 400 mg/h continuous infusion. Standard monitoring included arterial and central venous blood pressure, ECG with ST-segment analysis, transesophageal echocardiography, bladder temperature, pulse oximetry, end-tidal carbon dioxide, end-tidal volatile anesthetic concentration, respiratory mechanics, and urinary output. If aortic cross-clamping was performed, moderate hypothermia and cardioplegic arrest of the heart were induced with antegrade and/or retrograde and/or selective cold blood (Buckberg) or cold crystalloid (Custodiol) cardioplegic solution, as preferred by the surgeon. Before CPB initiation, an activated clotting time (ACT) greater than 480 seconds was achieved with unfractionated heparin administration (3 mg/kg) and was reversed with protamine in a 1 to 1 ratio after CPB weaning. Intraoperative myocardial protection was performed with cardioplegia or, as preferred by the anesthesiologist, with short-acting beta-blockade (esmolol given before aortic cross-clamping and/or added to the cardioplegic solution) and/or with volatile anesthetics during CPB.

Ventilatory management was based on a mean tidal volume of 8 ml/kg of Ideal Body Weight (IBW) with a Positive End-Expiratory Pressure (PEEP) of a minimum level of 5 cmH_2O applied to all

patients, unless contraindicated (e.g. dynamic overinflation, hemodynamic instability etc). The respiratory rate was titrated to maintain normocapnia. Before releasing aortic cross-clamping, a recruitment maneuver was routinely performed in all patients. Airway pressure was set to 40 cmH_2O for at least 10 seconds, with the double purpose of de-airing and re-expanding lung parenchyma. No ventilatory protective strategy during CPB was applied as a standard method. Ventilation was routinely discontinued during CPB.

After surgery, unconsciousness was maintained with continuous propofol infusion, and patients were transferred to the ICU. When hemodynamic stability with no significant bleeding, normothermia, and an adequate level of consciousness and pain control was achieved, weaning from mechanical ventilation was started. Post-operative pain relief was provided to all patients by intravenous acetaminophen and morphine. Chest physiotherapy was started as soon as possible for all patients after surgery. Non-invasive mechanical ventilation (NIMV) was initiated when clinically indicated to facilitate weaning from mechanical ventilation, and was continued in the ward following ICU discharge.

Supplementary material 3: The full logistic regression model

Preoperative model

Age (years), surgical diagnosis, coronaropathy (yes/no), ACEF score value[28] (%), BMI (kg/m^2), redo surgery (yes/no), elective surgery (yes/no), hypertension (yes/no), diabetes mellitus (yes/no), chronic obstructive pulmonary disease (yes/no), peripheral vasculopathy (yes/no), previous ictus or transitory ischemic attack (yes/no), preoperative history of smoking (yes/no), preoperative ejection fraction (%), preoperative serum creatinine (mg/dl), New York Heart Association class (from I to IV), ASA class (from I to V)

ACEF: Age, creatinine, ejection fraction.

Surgery model

Aortic cross-clamp (yes/no) and aortic cross-clamp time (minutes), blood-product administration during surgery (yes/no), inotropic support during surgery (yes/no), and use of Intra-Aortic Balloon Pump (IABP) (yes/no)

ICU model

Peak postoperative creatinine in ICU (mg/dl), the lowest hematocrit value in ICU (%), inotropic drugs after surgery (yes/no), postoperative IABP (yes/no), duration of mechanical ventilation (hours), postoperative renal replacement therapy (yes/no), postoperative Extracorporeal Membrane Oxygenator (ECMO) (yes/no), tracheostomy in ICU (yes/no), re-intubation in ICU (yes/no), postoperative pulmonary complication (PPC[14]) (yes/no), septic shock (yes/no, according to the Sepsis-3 consensus definition[27]), use of diuretic therapy (yes/no), blood-product administration during ICU stay (yes/no), PaO_2/FiO_2 ratio on ICU discharge.
